# Doxorubicin attenuates CHIP-guarded HSF1 nuclear translocation and protein stability to trigger IGF-IIR-dependent cardiomyocyte death

**DOI:** 10.1038/cddis.2016.356

**Published:** 2016-11-03

**Authors:** Chih-Yang Huang, Wei-Wen Kuo, Jeng-Fan Lo, Tsung-Jung Ho, Pei-ying Pai, Shu-Fen Chiang, Pei-Yu Chen, Fu-Jen Tsai, Chang-Hai Tsai, Chih-Yang Huang

**Affiliations:** 1 Translation Research Core, China Medical University Hospital, China Medical University, Taichung, Taiwan; 2 Department of Biological Science and Technology, China Medical University, Taichung, Taiwan; 3 Institute of Oral Biology, National Yang-Ming University, Taipei, Taiwan; 4 Chinese Medicine Department, China Medical University Beigang Hospital, Yunlin, Taiwan; 5 School of Chinese Medicine, China Medical University, Taichung, Taiwan; 6 Division of Cardiology, China Medical University Hospital, Taichung, Taiwan; 7 Cancer Center, China Medical University Hospital, Taichung, Taiwan; 8 Department of Pathology, China Medical University Hospital, Taichung, Taiwan; 9 Department of Healthcare Administration, Asia University, Taichung, Taiwan; 10 Graduate Institute of Basic Medical Science, China Medical University, Taichung, Taiwan; 11 Department of Health and Nutrition Biotechnology, Asia University, Taichung, Taiwan

## Abstract

Doxorubicin (DOX) is one of the most effective antitumor drugs, but its cardiotoxicity has been a major concern for its use in cancer therapy for decades. Although DOX-induced cardiotoxicity has been investigated, the underlying mechanisms responsible for this cardiotoxicity have not been completely elucidated. Here, we found that the insulin-like growth factor receptor II (IGF-IIR) apoptotic signaling pathway was responsible for DOX-induced cardiotoxicity via proteasome-mediated heat shock transcription factor 1 (HSF1) degradation. The carboxyl-terminus of *Hsp70* interacting protein (CHIP) mediated HSF1 stability and nuclear translocation through direct interactions via its tetratricopeptide repeat domain to suppress IGF-IIR expression and membrane translocation under physiological conditions. However, DOX attenuated the HSF1 inhibition of IGF-IIR expression by diminishing the CHIP–HSF1 interaction, removing active nuclear HSF1 and triggering HSF1 proteasomal degradation. Overexpression of CHIP redistributed HSF1 into the nucleus, inhibiting IGF-IIR expression and preventing DOX-induced cardiomyocyte apoptosis. Moreover, HSF1A, a small molecular drug that enhances HSF1 activity, stabilized HSF1 expression and minimized DOX-induced cardiac damage *in vitro* and *in vivo*. Our results suggest that the cardiotoxic effects of DOX result from the prevention of CHIP-mediated HSF1 nuclear translocation and activation, which leads to an upregulation of the IGF-IIR apoptotic signaling pathway. We believe that the administration of an HSF1 activator or agonist may further protect against the DOX-induced cell death of cardiomyocytes.

Cardiotoxicity occurs frequently in patients with tumors treated with anthracyclines, such as doxorubicin (DOX). As a result of this irreversible cardiotoxicity, the clinical use of DOX remains significantly limited. The cardiotoxicity of DOX involves increased oxidative stress, apoptosis and a direct effect on DNA synthesis in cardiomyocytes;^1, 2, 3^ however, the detailed mechanism of DOX-induced heart failure is not well established.

Insulin-like growth factor receptor II (IGF-IIR) is a multifunctional protein that has a vital role in the regulation of cardiac development, growth and survival.^4, 5^ Several lines of evidence indicate that IGF-IIR may participate in pathological processes of heart failure, such as hypertrophy and end-stage heart failure.^6, 7, 8, 9^ Our previous findings clearly indicate that the IGF-II/ IGF-IIR signaling pathway is implicated in the stages of progressive of heart failure, such as pathological hypertrophy,^10, 11^ myocardial remodeling fibrosis^5^ and cardiomyocyte apoptosis.^12, 13^ Upregulation of IGF-IIR expression was shown to be involved in hypertension-induced cardiomyocyte apoptosis, and its expression is correlated with the progression of cardiomyocyte apoptosis in hypertensive rat hearts.^14^ Therefore, it is crucial to uncover the regulatory mechanisms controlling IGF-IIR gene expression, as regulating this gene may be a good strategy for both the protection against cardiomyocyte death and the prevention of the progression of heart failure.

Recently, our studies have revealed the detailed mechanisms for regulating IGF-IIR gene expression.^15, 16^ Our findings clearly indicate that heat shock transcription factor 1 (HSF1) acts as a cardioprotective factor that controls the IGF-IIR expression responsible for stress response in cardiomyocytes.^16^ However, the detailed mechanism of HSF1 activation in the control of IGF-IIR expression is not well established. Previous studies have shown that the activation of HSF1 is tightly controlled by diverse mechanisms.^17^ It has been reported that several molecular chaperones, including Hsp70, Hsp90 and Hsp40, have roles in the attenuation phase of HSF1 activation,^18, 19, 20^ suggesting that chaperone proteins can bind to HSF1 and regulate its activation.

The co-chaperone carboxyl-terminus of *Hsp70* interacting protein (CHIP) possesses a tetratricopeptide repeat (TPR) domain at its N terminus for interactions with Hsp70 and a U-box domain at its C terminus for ubiquitination.^21^ CHIP has been shown to have a protective role by targeting misfolded or damaged proteins that are associated with the pathologies of neurodegenerative^21, 22^ and heart diseases.^23, 24, 25^ Moreover, CHIP can directly interact with the N-terminal domain of HSF1, facilitating HSF1 nuclear translocation and activation to protect against stress-induced apoptosis.^26, 27^ Although CHIP and HSF1 have been shown to be responsible for oxidative stress-induced cell death in neurons and cardiomyocytes,^28, 29^ the detailed mechanism of how CHIP protects cells from DOX-induced oxidative stress has not been well elucidated.

These studies suggest that cardiomyocyte death because of DOX may be a result of IGF-IIR-induced apoptosis signaling. Under physiological conditions, CHIP maintains HSF1 stability through direct interactions between the N-terminal TPR domain of CHIP and HSF1. This interaction results in HSF1 nuclear translocation and activation, which protects against cardiomyocyte death via the suppression of IGF-IIR expression. However, DOX administration triggers ubiquitin-proteasome degradation, which disrupts the CHIP–HSF1 interaction and leads to HSF1 instability. Eventually, HSF1 degradation results in IGF-IIR upregulation, triggering cardiomyocyte death.

## Results

### IGF-IIR contributed to the DOX-induced apoptotic pathway in cardiomyocytes

**Figure 1:**
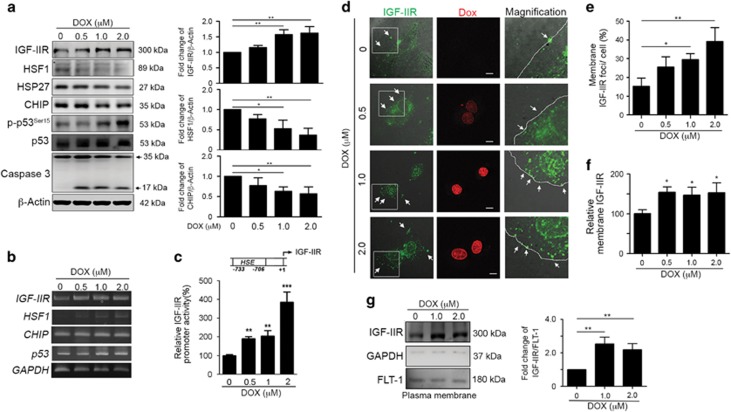
DOX markedly upregulated IGF-IIR expression and induced its translocated into the membrane. (**a**) H9c2 cells were treated with different concentrations of DOX for 24 h. Cells were harvested and analyzed via immunoblotting. Quantification of the results is shown right (*n*=3). *P<0.05 and **P<0.01. (**b**) H9c2 cells were treated with DOX for 24 h. RNA was extracted and analyzed via RT-PCR. (**c**) H9c2 cells were transfected with pGL3-IGF-IIR (-1200~+1) for 24 h and then treated with DOX for 24 h. Cells were harvested and analyzed for luciferase activity. **P<0.01 and ***P<0.001. (**d**) H9c2 cells were treated with DOX for 24 h. Cells were fixed with 4% paraformaldehyde and stained with antibodies against IGF-IIR. The IGF-IIR foci on the surface of H9c2 cells were observed with confocal microscopy. (**e**) One hundred H9c2 cells were counted and statistically analyzed. *P<0.05 and **P<0.01. (**f**) H9c2 cells were treated with DOX for 24 h. Membrane IGF-IIR proteins were directly estimated via ELISA. *P<0.05. (**g**) H9c2 cells were treated with different concentrations of DOX for 24 h. Quantification of the results is shown right (*n*=3). **P<0.01. Cells were harvested and membrane proteins were isolated. The amount of IGF-IIR in the membranes was estimated via immunoblotting. These data were obtained from at least three independent experiments and values represent the means ±S.D.

Our previous studies have demonstrated that activated membrane-bound IGF-IIR recruits G*α*q to induce caspase-3-dependent apoptosis in response to injuries leading to the progression of heart failure, such as spontaneous hypertension-induced and abdominal aorta ligation-induced cardiac injury.^14, 16^ Therefore, we attempted to clarify whether IGF-IIR was implicated in zDOX-induced heart failure. With elevated concentrations of DOX, p53 and caspase-3 activation was markedly increased to induce apoptosis (Figure 1a).^30, 31, 32^ Interestingly, IGF-IIR expression was strongly upregulated, implying that the IGF-IIR signaling pathway may be involved in DOX-induced cardiomyocyte death.

Our earlier studies demonstrated that the expression of IGF-IIR is negatively regulated by HSF1.^16^ Notably, HSF1 and its downstream protein Hsp27 were significantly decreased, suggesting that DOX may enhance IGF-IIR expression via reducing HSF1 expression. Indeed, we observed that *IGF-IIR* mRNA and promoter activities were gradually upregulated after exposure to DOX (Figures 1b and c). But *HSF1* mRNA was increased after DOX administration. These results suggest that DOX-induced cardiac cell death may result from the loss or dysregulation of HSF1, resulting in IGF-IIR activation.

We then estimated whether membrane-bound IGF-IIR, which was reported to participate in cardiomyocyte apoptosis,^16^ was increased by DOX. IGF-IIR markedly translocated to the plasma membrane in DOX exposure but only a few IGF-IIR foci were detected at the plasma membrane under physiological condition (Figures 1d and e). Similarly, the amount of membrane-bound IGF-IIR was significantly upregulated with increases in DOX concentrations, based on ELISA and fractionation analyses (Figures 1f and g).

**Figure 2:**
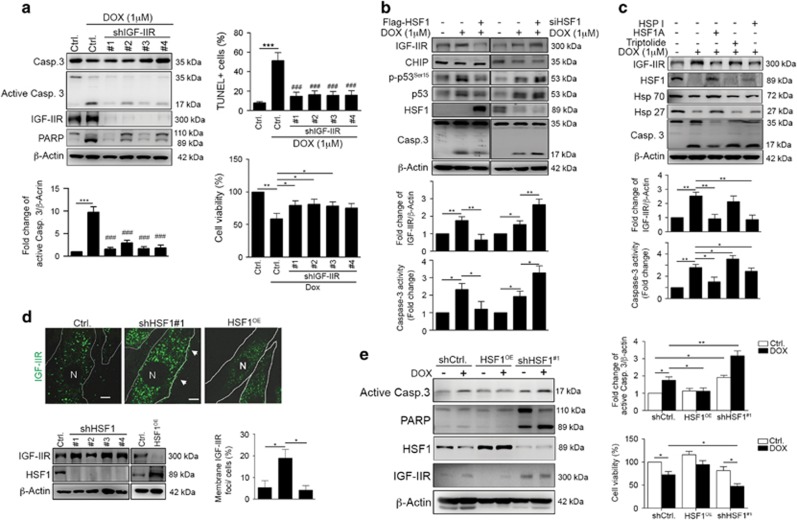
IGF-IIR is implicated in DOX-induced cardiomyocyte apoptosis, whereas HSF1 prevented DOX-induced cell death by suppressing IGF-IIR. (**a**) H9c2-IGF-IIR^KD^ stable cells were treated with 1* μ*M DOX for 24 h and harvested for immunoblot analysis. Quantification of active caspase-3 levels, TUNEL^+^ cardiomyocytes (%TUNEL^+^ cells as followed: 7.89±1.21, 51.43±8.32, 14.65±4.20, 16.32±3.91, 15.64±3.99, 15.9±4.53) and cell viability are shown (*n*=3). *P<0.05, **P<0.01 and ***P<0.001. ^###^ represents a significant decrease relative to the DOX-treated cells *P*<0.001. (**b**) H9c2 cells were transfected with Flag-HSF1 and HSF1 siRNA for 24 h and then treated with 1 *μ*M DOX for 24 h. Cells were harvested and analyzed via immunoblotting. Quantification of IGF-IIR levels and caspase-3 activity are shown below (*n*=3). *P<0.05 and **P<0.01. (**c**) H9c2 cells were pretreated with 10 *μ*M HSP1 (Hsp70 inhibitor), 10 *μ*M HSF1A (HSF1 activator) and 1 *μ*M triptolide (HSF1 inhibitor) for 2 h and then co-treated with DOX for 24 h. Cells were harvested and analyzed via immunoblotting. Quantification of IGF-IIR levels and caspase-3 activity are shown below (*n*=3). *P<0.05 and **P<0.01. (**d**) H9c2 cells were infected with lentiviruses carrying shRNA against HSF1 and lentiviruses carrying full-length HSF1. After infection, cells were selected with puromycin to generate the H9c2-HSF1^KD^ cells and H9c2 cells stably expressing HSF1 (H9c2-HSF1^OE^). Cells were analyzed via immunoblotting and confocal microscopy. One hundred fifty cells were counted and statistically analyzed. The percentages indicate the number of cells for with we observed membrane IGF-IIR foci. Quantification of IGF-IIR levels and caspase-3 activity are shown below (*n*=3). *P<0.05 and **P<0.01. (**e**) H9c2-HSF1^KD^ and H9c2-HSF1^OE^ stable cells were treated with DOX for 24 h and analyzed via immunoblotting. The apoptosis markers were estimated via immunoblotting. Quantification of active caspase-3 levels and cell viability are shown right (*n*=3). *P<0.05 and **P<0.01. These data were obtained from at least three independent experiments and values represent the means±S.D.

To determine if IGF-IIR is necessary for DOX-induced cardiomyocyte apoptosis, we utilized lentiviral infection carrying specific IGF-IIR-targeting small hairpin RNAs (shRNAs) to silence its expression. Administration of DOX to these IGF-IIR-deficient cells showed that the cleavage of caspase-3 and its downstream substrate PARP were significantly decreased, indicating that IGF-IIR is responsible for DOX-induced cardiomyocyte apoptosis (Figure 2a). Moreover, the TUNEL+ cardiomyocytes were clearly reduced and cell viability was retained in IGF-IIR-deficient cells after challenged with DOX (Figure 2a). Taken together, these results show that membrane-bound IGF-IIR-activated apoptotic signaling has a critical role in DOX-induced cardiomyocyte death.

### Dysregulation of HSF1 is responsible for IGF-IIR-mediated cardiomyocyte apoptosis during DOX-induced cardiotoxicity

HSF1 has been identified to suppress IGF-IIR expression to protect cardiomyocytes,^12, 13, 14, 16^ we assumed that the dysregulation of the HSF-IGF-IIR participates in DOX-induced heart failure. Indeed, highly expressed HSF1 protects cardiomyocytes from apoptosis by decreasing the DOX-induced IGF-IIR upregulation, caspase-3 cleavage and caspase-3 activity (Figure 2b). Conversely, knockdown of HSF1 significantly enhanced DOX-stimulated IGF-IIR expression and caspase-3 cleavage, suggesting that HSF1-mediated IGF-IIR suppression is required for and sufficient to protect cardiomyocytes from DOX-induced cardiotoxicity.

We further investigated whether the HSF1 influenced DOX-induced cardiomyocyte apoptosis using a well-characterized small molecule HSF1-specific activator and inhibitor, HSF1A^33^ and triptolide,^34, 35^ respectively. Notably, HSF1A significantly inhibited IGF-IIR upregulation, caspase-3 cleavage and caspase-3 activity under DOX treatment (Figure 2c). However, triptolide slightly elevated DOX-induced IGF-IIR expression and caspase-3 activation, demonstrating that HSF1 may be responsible for DOX-induced cardiomyocyte apoptosis. We then treated cells with Hsp70 inhibitor HSP1 (refs 38, 39) to activate the DNA-binding activity and transactivation capacity of HSF1.^17, 36^ Treatment with HSP1 indeed enhanced HSF1 transcription activity to upregulate Hsp27 expression (Figure 2c). HSP1 further suppressed IGF-IIR upregulation under DOX treatment, implying that Hsp70 may be involved in HSF1-regulated IGF-IIR expression via modulating the transactivation capacity of HSF1. Moreover, HSF1 deficiency led to the upregulation of both expressed and membrane-bound IGF-IIR (Figure 2d). Constitutively expressed HSF1 markedly inhibited IGF-IIR expression and alleviated the DOX-induced cardiotoxicity, preventing cardiomyocyte apoptosis and increasing cell viability (Figure 2e). Taken together, these results indicate that the dysregulation of HSF1-mediated IGF-IIR suppression contributes to DOX-induced cardiotoxicity.

### CHIP directly interacted with HSF1, whereas DOX diminished this interaction

**Figure 3:**
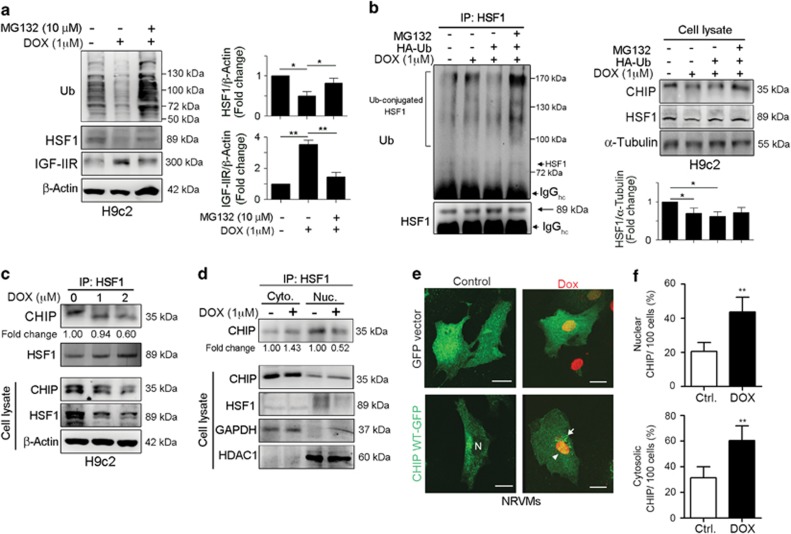
DOX induced HSF1 instability by triggering proteasome degradation activities. (**a**) H9c2 cells were treated with DOX for 18 h and then co-treated with 10 *μ*M MG132 for 6 h. Cells were harvested and analyzed via immunoblotting. Quantification of HSF1 and IGF-IIR level are shown right (*n*=3). *P<0.05 and **P<0.01. (**b**) H9c2 cells were transfected with HA-ubiquitin for 24 h and then treated with DOX for 18 h and 10 *μ*M MG132 for 6 h simultaneously. Cells were harvested and analyzed via immunoprecipitation and immunoblotting. Immunoprecipitation of 500* μ*g of the cell lysates was performed with antibodies against HSF1, and the samples were analyzed via immunoblotting. Quantification of HSF1 level are shown below (*n*=3). *P<0.05. (**c**) H9c2 cells were treated with 1 or 2 *μ*M DOX for 24 h and harvested for immunoprecipitation. Immunoprecipitation was carried out on 500* μ*g of the cell lysates with HSF1 antibodies, and the samples were analyzed via immunoblotting. (**d**) H9c2 cells were treated with 1 *μ*M DOX for 24 h and fractionated into cytosolic and nuclear proteins. Immunoprecipitation was carried out on 300* μ*g of the cytosolic and 500* μ*g of the nuclear proteins with HSF1 antibodies, and the samples were analyzed via immunoblotting. Identical amounts of cytosolic and nuclear proteins were estimated via immunoblotting. (**e**) NRVMs were transfected with GFP-CHIP WT or GFP alone for 24 h and treated with DOX for 24 h. The localization of GFP-CHIP was observed using confocal microscopy. One hundred cells were counted and statistically analyzed. Quantification of cytosolic and nuclear CHIP are shown in (**f**). **P<0.01. These data were obtained from at least three independent experiments and values represent the means±S.D.

Recent studies have excitingly revealed that DOX can activate ubiquitin-proteasome system (UPS)-mediated proteolysis on pivotal cardiac transcription factors and survival factors in cardiomyocytes.^37, 38^ We observed that DOX clearly activated UPS-mediated proteolysis, whereas the proteasome inhibitor MG132 significantly inhibited proteolysis and led to the accumulation of ubiquitin-conjugated proteins, which is an important feature of proteasomal inhibition (Figure 3a). DOX treatment slightly enhanced the level of Ub-conjugated HSF1; however, the overexpression of ubiquitin significantly decreased the level of Ub-conjugated HSF1, suggesting that ubiquitin accelerated the DOX-induced degradation of HSF1. MG132 alleviated the DOX-induced degradation of HSF1 and led to the accumulation of Ub-conjugated HSF1 proteins (Figure 3b). These results indicate that HSF1 is the target of DOX in inducing cardiomyocytes apoptosis through the IGF-IIR signaling pathway.

CHIP regulates the stress-chaperone response through inducing the trimerization and transcriptional activation of HSF1 by facilitating HSF1 nuclear translocation in protecting against apoptosis.^27^ Based on immunoprecipitation analysis, we found that CHIP directly interacted with HSF1. This interaction was markedly diminished with elevated DOX concentrations (Figure 3c). Moreover, the interaction was significantly detected in the nuclear fraction under normal conditions (Figure 3d). DOX altered the nuclear CHIP–HSF1 interaction, implying that CHIP may be facilitating HSF1 nuclear translocation through direct interactions under physiological conditions; however, DOX treatment diminished this translocation. Consistent with this, confocal microscopy images showed that GFP-CHIP was primarily localized in the cytoplasm, with a lower nuclear GFP-CHIP distribution in primary neonatal rat ventricular myocytes (NRVMs; Figures 3e and f). However, DOX treatment influenced this distribution, resulting in GFP-CHIP being relocalized into the nucleus and forming aggregates in the cytoplasm.

**Figure 4:**
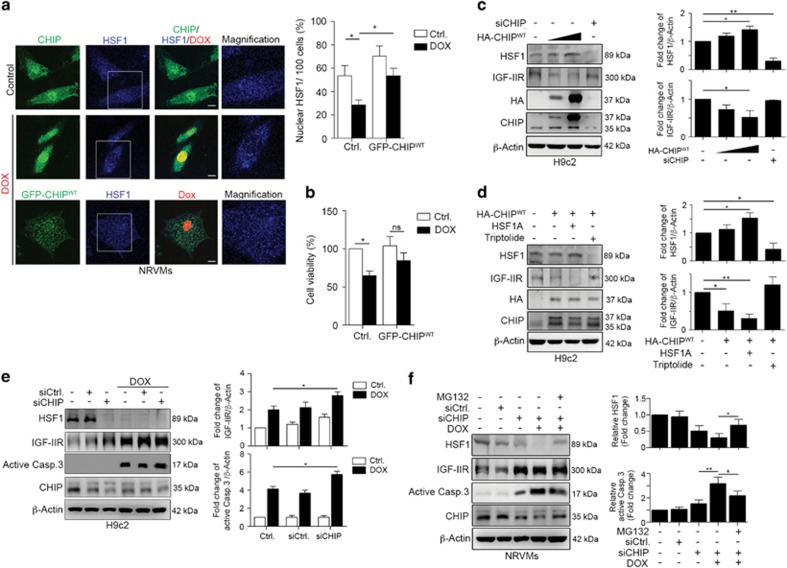
CHIP maintained HSF1 stability and facilitated its nuclear translocation and activation. (**a**) NRVMs cells were transfected with 5* μ*g and GFP-CHIP WT. Then, normal and transfected cells were treated with DOX for 24 h and analyzed using confocal microscopy. The nuclear CHIP and cytosolic CHIP foci were clearly observed, whereas nuclear HSF1 was excluded after DOX treatment. One hundred cells were counted and statistically analyzed. Quantification of nuclear CHIP is shown right. *P<0.05. (**b**) NRVMs cells were transfected with 5* μ*g and GFP-CHIP WT. Then, normal and transfected cells were treated with DOX for 24 h and analyzed using MTT assay. Quantification of cell viability is shown right (*n*=3). *P<0.05. (**c**) H9c2 cells were transfected with 5* μ*g or 10* μ*g HA-CHIP WT and 10 nM CHIP siRNA for 48 h. HSF1 and IGF-IIR expression levels were analyzed via immunoblotting. Quantification of HSF1 and IGF-IIR levels is shown right (*n*=3). *P<0.05 and **P<0.01. (**d**) H9c2 cells were transfected with 5* μ*g HA-CHIP WT for 24 h and then co-treated with 1 *μ*M triptolide and 10 *μ*M HSF1A for 24 h. HSF1 and IGF-IIR expression levels were analyzed via immunoblotting. Quantification of HSF1 and IGF-IIR levels is shown right (*n*=3). *P<0.05 and **P<0.01. (**e**) H9c2 cells were transfected with 10 nM scrambled or CHIP siRNA. Then, transfected cells were treated with 1 *μ*M DOX for 24 h. HSF1, IGF-IIR and caspase-3 were estimated via immunoblotting. Quantification of IGF-IIR and active caspase-3 levels is shown right (*n*=3). *P<0.05. (**f**) Primary NRVMs were transfected with 10 nM scrambled or CHIP siRNA. Then, transfected cells were treated with 1 *μ*M DOX for 18 h and 10 *μ*M MG132 for 6 h. HSF1, IGF-IIR and caspase-3 were estimated via immunoblotting. Quantification of HSF1 and active caspase-3 levels is shown right (*n*=3). *P<0.05 and **P<0.01. These data were obtained from at least three independent experiments and values represent the means±S.D.

Interestingly, CHIP was wildly distributed in the cytoplasm and nucleus, whereas HSF1 was mainly located in the nucleus in NRVMs (Figure 4a and Supplementary Figure S1A). However, the location of HSF1 was significantly altered in the presence of DOX, suggesting that the nuclear exclusion of HSF1 may be due to its dissociation from CHIP (Figure 4a and Supplementary Figure S1B). These results indicate that CHIP facilitated HSF1 nuclear translocation and activation via their direct interaction to protect against stresses, such as DOX exposure.

### CHIP is necessary for HSF1 stability and activation to prevent DOX-induced cardiomyocyte cell death

Moreover, these enforced expression levels of CHIP clearly alleviated DOX-induced cell death (Figure 4b) and slightly stabilized HSF1 expression, whereas the knockdown of CHIP significantly decreased HSF1 expression (Figure 4c), implying that CHIP may stabilize HSF1 via direct interactions to inhibit the IGF-IIR-induced apoptotic signaling pathway. We further treated with HSF1A and triptolide to clarify whether CHIP-mediated HSF1 activity to inhibit IGF-IIR expression (Figure 4d). Notably, the combination of CHIP and HSF1A clearly inhibited IGF-IIR expression. Conversely, triptolide upregulated IGF-IIR expression even with the overexpression of CHIP, clarifying that IGF-IIR expression is negatively regulated by HSF1, which is stabilized by CHIP (Figure 4d).

We then examined whether CHIP deficiency enhanced DOX-induced apoptosis via the IGF-IIR signaling pathway. Consistent with earlier results, CHIP deficiency led to HSF1 instability, which in turn upregulated IGF-IIR expression (Figure 4e and Supplementary Figure S2). The upregulation of IGF-IIR increased cardiomyocyte sensitivities to DOX, suggesting that the extent of IGF-IIR expression may determine cardiomyocyte apoptosis in response to stresses such as DOX exposure. Knockdown of CHIP led to a decrease in HSF1 and an increase in IGF-IIR expression, which resulted in a greater sensitivity to stress. Similarly, we found that the absence of CHIP clearly reduced HSF1 expression and increased IGF-IIR expression in NRVMs (Figure 4f). Moreover, MG132 rescued HSF1 expression and inhibited IGF-IIR and caspase-3 activation. These results show that the presence of CHIP stabilizes HSF1 and inhibits IGF-IIR-induced apoptosis is more critical in NRVMs.

### CHIP directly interacted with HSF1 via its TPR domain to stabilize its expression to protect against DOX exposure

**Figure 5:**
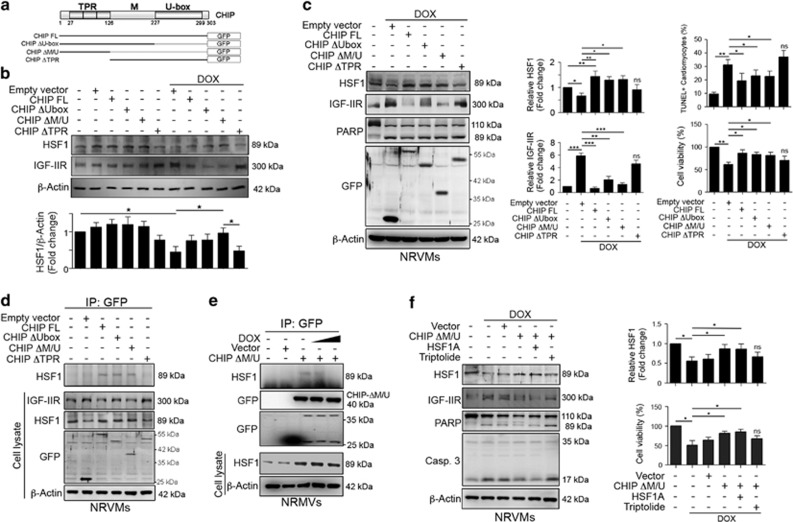
CHIP directly interacted with HSF1 through its TPR domain to maintain its expression against DOX-induced cardiomyocyte apoptosis. (**a**) The schematic diagram of CHIP deletion mutations, which were conjugated with green fluorescent protein (GFP). (**b**) H9c2 cells were transfected with different CHIP deletion mutant constructs for 24 h and then treated with 1 *μ*M DOX for 24 h. HSF1 and IGF-IIR were estimated via immunoblotting. Quantification of HSF1 level are shown below (*n*=3). *P<0.05. (**c**) Primary NRVMs were transfected with different CHIP deletion mutant constructs for 24 h and then treated with 1 *μ*M DOX for 24 h. HSF1, IGF-IIR and PARP were estimated via immunoblotting. Quantification of HSF1 level, IGF-IIR level, TUNEL+ cardiomyocytes (%TUNEL^+^ cells as followed: 9.74±1.31, 31.32±3.75, 19.43±5.42, 23.16±4.21, 22.65±3.98, 37.14±4.86) and cell viability is shown right (*n*=3). *P<0.05, **P<0.01 and ***P<0.001. (**d**) Primary NRVMs were transfected with different CHIP deletion mutant constructs for 48 h. Cell lysates (500* μ*g) were immunoprecipitated with antibodies against GFP and analyzed via immunoblotting. (**e**) Primary NRVMs were transfected with CHIP-ΔM/U-GFP for 24 h and treated with 1 or 2 *μ*M DOX for 24 h. Cell lysates (500* μ*g) were immunoprecipitated with antibodies against GFP and analyzed via immunoblotting. (**f**) Primary NRVMs were transfected with CHIP-ΔM/U-GFP for 24 h. Cells were then treated 10 *μ*M HSF1A or 1 *μ*M triptolide with 1 *μ*M DOX for 24 h. The expression of HSF1, PARP, caspase-3 and IGF-IIR were evaluated via immunoblotting. Quantification of HSF1 level and cell viability is shown right (*n*=3). *P<0.05. These data were obtained from at least three independent experiments and values represent the means±S.D.

Previous studies showed that CHIP may act as a chaperone to modulate protein stability^22^ or prevent protein degradation through noncanonical ubiquitination.^39^ Therefore, we generated CHIP deletion mutants, which contained different fragments of CHIP that were conjugated with GFP as shown in Figure 5a. We found that CHIP-WT slightly stabilized HSF1 and mediated IGF-IIR expression (Figure 5b). Similarly, CHIP-ΔM/U and CHIP-ΔU-box domains retained HSF1 activity and suppressed IGF-IIR expression. Moreover, treatment with DOX resulted in HSF1 degradation. CHIP-WT, CHIP-ΔM/U and CHIP-ΔU-box rescued HSF1 expression and repressed IGF-IIR expression, with CHIP-ΔU-box fragment having the greatest effect. Similarly, CHIP-ΔU-box fragment had the better impacts on the suppression of DOX-induced cardiomyocytes death in primary NRVMs (Figure 5c and Supplementary Figure S3A).

We then examined which fragment of CHIP determined its interaction with HSF1 using an immunoprecipitation assay. As shown in Figure 5d, CHIP-FL, CHIP-ΔU-box and CHIP-ΔM/U interacted with HSF1 based on immunoprecipitation. However, CHIP-ΔTPR did not interact with HSF1, which suggests that CHIP interacts with HSF1 *via* its TPR domain. Notably, the CHIP-ΔM/U fragment directly interacted with HSF1, and this interaction gradually decreased after DOX treatment (Figure 5e). Furthermore, HSF1A significantly enhanced the ability of CHIP-ΔM/U to inhibit IGF-IIR expression, reduce caspase-3 activation and retained cell viability (Figure 5f and Supplementary Figure S3B). However, triptolide reduced the protective effect of CHIP-ΔM/U and led to caspase-3 activation. These results clearly indicate that CHIP stabilized HSF1 expression through direct interactions via its TPR domain. However, DOX resulted in the dissociation of CHIP and HSF1, which triggered the induction of the IGF-IIR apoptotic signaling pathway.

### The Hsp70-binding activity of CHIP is not required for improving HSF1 stability

**Figure 6:**
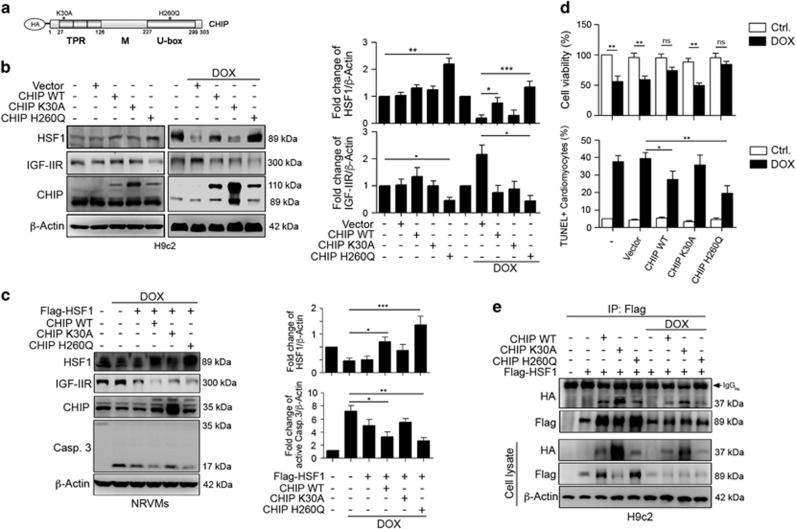
Hsp70-binding activity was not required for CHIP to interact with HSF1. (**a**) The schematic diagram of the CHIP point mutations, which were conjugated with hemagglutinin (HA). (**b**) H9c2 cells were transfected with HA-CHIP WT, HA-CHIP-K30A (Hsp70-binding activity defect mutant), and HA-CHIP-H260Q (E3 ligase activity defect mutant) for 24 h and then treated with 1 *μ*M DOX for 24 h. HSF1 and IGF-IIR were estimated via immunoblotting. Quantification of HSF1 and IGF-IIR level is shown right (*n*=3). *P<0.05, **P<0.01 and ***P<0.001. (**c**) Primary NRVMs were transfected with HA-CHIP WT, HA-CHIP-K30A, HA-CHIP-H260Q and Flag-HSF1 together for 24 h and then treated with 1 *μ*M DOX for 24 h. HSF1, IGF-IIR and caspase-3 were estimated via immunoblotting. Quantification of HSF1 and active Caspase-3 level is shown right (*n*=3). *P<0.05, **P<0.01 and **P<0.001. (**d**) Primary NRVMs cells were transfected with HA-CHIP WT, HA-CHIP-K30A, and HA-CHIP-H260Q for 24 h and then treated with 1 *μ*M DOX for 24 h. Quantification of cell viability and TUNEL^+^ cardiomyocytes is shown right (*n*=3). The percentage of TUNEL+ cardiomyocytes is shown as followed (Ctrl. *versus* Dox: 5.12±0.1 *versus* 37.53±3.54; 4.32±0.47 *versus* 39.53±3.32; 5.32±0.64 *versus* 27.53±4.76; 3.43±0.54 *versus* 35.75±5.76; 4.65±0.87 *versus* 19.53±4.31).*P<0.05 and **P<0.01. (**e**) H9c2 cells were transfected with HA-CHIP WT, HA-CHIP-K30A, HA-CHIP-H260Q and Flag-HSF1 for 24 h. The same amounts of cell lysates from each treatment were immunoprecipitated with antibodies against Flag and were analyzed via immunoblotting. These data were obtained from at least three independent experiments and values represent the means ±S.D.

As TPR domain of CHIP is necessary for its co-chaperone activities via interactions with the Hsp70 and Hsp90 molecular chaperones,^21^ we attempted to identify whether the binding activity of CHIP and Hsp70 is essential for the effects of CHIP in stabilizing HSF1 expression and preventing cardiomyocyte apoptosis. Western blotting results showed that enforced expression of CHIP-WT and CHIP-H260Q (lacking E3 ligase activity) slightly increased HSF1 expression under normal conditions (Figures 6a and b). Moreover, CHIP-H260Q remarkably retained

HSF1 expression after DOX administration, which maintains cell viability and reduces TUNEL^+^ cardiomyocytes (Figures 6c and d). These results indicated that CHIP regulated HSF1 stability via its TPR domain to mediate IGF-IIR. We then identify whether the interaction between Hsp70 and CHIP is necessary for the CHIP-mediated HSF1 stability. Surprisingly, these mutant forms of CHIP still remained able to bind to HSF1, and this interaction was decreased with exposure to DOX (Figure 6e). These results indicate that Hsp70 was not necessary for CHIP and HSF1 to interact. Taken together, these results clearly indicate that CHIP bound to and stabilized HSF1 via its TPR domain and that Hsp70 is not involved in this interaction under physiological conditions. Once cells suffered stress, such as DOX exposure, CHIP probably lost its protective role in maintaining HSF1 stability.

### Challenge with DOX led to heart failure, whereas HSF1A ameliorated DOX-induced heart failure *in vivo*

**Figure 7:**
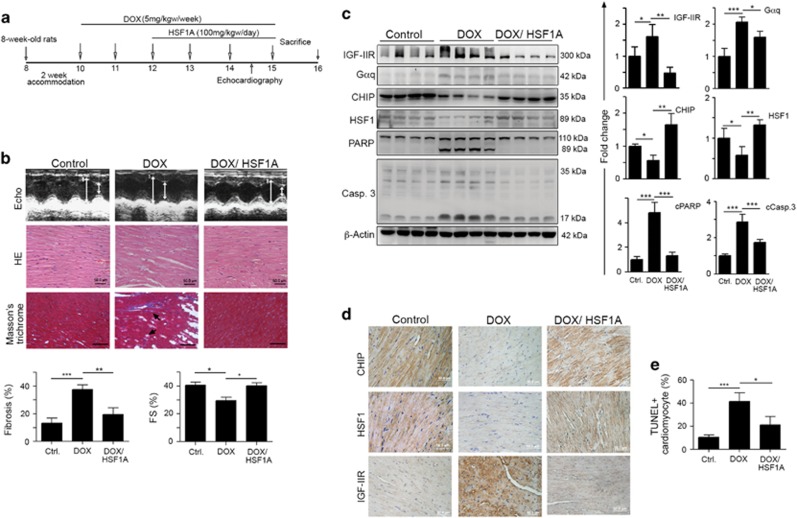
DOX impaired CHIP to destabilize HSF1, activating the IGF-IIR apoptotic signaling pathway *in vivo.* (**a**) The schematic procedure of DOX and HSF1A administration. (**b**) After treatment with DOX and supplementation with HSF1A, the cardiac characteristics of different groups were estimated via echocardiography, hematoxylin–eosin staining (HE staining). Echocardiographic assessments of the cardiovascular structure and function of these three groups: control rat group (control), DOX-treated rat group (DOX) and DOX-treated rats supplemented with HSF1A group (DOX/HSF1A). HE staining of slides of heart ventricles. Cell nuclei are stained blue, and other structures are stained pink. The extent of cardiac fibrosis was estimated via Masson's trichrome. Quantification of cardiac fibrosis in left ventricular heart (*n*=4 per group) and FS (FS%, *n*=5 per group) is shown below. *P<0.05 and ***P<0.001. (**c**) The left ventricles of hearts were excised and homogenized. The cell lysates were quantified and analyzed via immunoblotting. The expression of the IGF-IIR signaling pathway protein and its downstream effector G*α*q and the expression of the apoptosis marker caspase-3 and its downstream substrate PARP, as well as that of CHIP and HSF1, were estimated via immunoblotting. Quantification of the results is shown right (*n*=4 per group). *P<0.05, **P<0.01 and ***P<0.001. (**d**) The expression of CHIP, HSF1, IGF-IIR and TUNEL^+^ cardiomyocytes (Ctrl.: 10.43±2.14; Dox: 41.64±7.43; Dox+HSF1A: 21.32±7.32,) were evaluated by immunohistochemistry (IHC) and TUNEL assay. Quantification of TUNEL^+^ cardiomyocytes from each group is shown in (**e**) (*n*=4 per group). These data were obtained from at least three independent experiments and values represent the means ±S.D.

We attempted to confirm that DOX reduced the effect of CHIP in stabilizing HSF1, leading to an increase in IGF-IIR-induced cardiomyocyte apoptosis *in vivo*. We challenged WKY rats with DOX (accumulated dose: 30 mg/kgw), and DOX combined with HSF1A (100 mg/kgw/day) (Figure 7a). After the administration of DOX, echocardiography was performed to analyze heart function (Figure 7b and Table 1). The cardiac functions in the DOX-treated group were significantly lower than those in the control group. The fractional shortening (FS) and ejection fraction (EF), parameters of cardiac function, were strongly reduced in DOX-treated rats. Interventricular septum at diastole (IVSd), left ventricular posterior wall thickness at diastole (LVPWd), internal dimension at diastole of the left ventricle (LVIDd) and internal dimension at systole of the left ventricle (LVIDs) were also decreased after DOX treatment. Interestingly, supplementation with HSF1A significantly elevated cardiac functions back to the levels of the control group. HSF1A has been shown to stimulate human HSF1 nuclear translocation, elevate protein chaperone expression and ameliorate protein misfolding and cell death in a neurodegenerative disease model.^33^ The echocardiographic results showed that HSF1A also alleviated DOX-induced failures in cardiac function. FS was clearly rescued in DOX-treated group after supplement with HSF1A.

We found that the prominent cardiac characteristics of the DOX-treated rats were significantly reduced relative to those of the control rats. However, in the HSF1A-administered group, the cardiac characteristics were found to remain relatively stable (Table 2). The whole heart weight (WHW) and left ventricular weight (LVW) were not different from the control group. However, LVW/WHW and the LVW/tibia length ratio in the DOX-treated group were decreased when compared with those in the control group. Surprisingly, relative to the DOX-treated rats, supplementation with HSF1A resulted in a retention of normal cardiac characteristics and appeared to alleviate the DOX-induced cardiac injuries. This staining showed that the ventricular myocardium in the control group had a normal architecture and a normal interstitial space (Figure 7b). Abnormal myocardial architecture, increased interstitial space, minor fibrosis and increased collagen deposition were all observed in the DOX-treated group but not in those also receiving HSF1A supplementation. Collagen accumulation in the DOX-treated rat hearts was clearly increased, indicating that cardiac fibrosis had occurred, whereas HSF1A administration reduced the extent of cardiac fibrosis (Figure 7b).

We then isolated left ventricular heart tissues to analyze the protein expression by immunoblotting. These results showed that IGF-IIR and its downstream effector G*α*q were clearly upregulated and that caspase-3 and its substrate PARP were significantly activated and that CHIP and HSF1 were obviously decreased in DOX-treated heart tissues. As expected, in the DOX-treated group supplemented with HSF1A, these proteins were relatively stable and similar to the levels in the control group heart tissues (Figure 7c). Immunohistochemistry staining showed that CHIP and HSF1 expression levels were reduced after DOX challenge, whereas IGF-IIR was increased. In contrast, supplementation with HSF1A significantly rescued CHIP and HSF1 expression and clearly suppressed IGF-IIR expression (Figure 7d). Moreover, TUNEL^+^ cardiomyocytes induced by DOX were remarkably reduced in supplementation with HSF1A (Figure 7e). Taken together, these results show that CHIP acted to protect cardiomyocytes via the stabilization of HSF1, protecting against apoptosis under physiological conditions, whereas the ability of CHIP to stabilize HSF1 was reduced in response to stresses, such as DOX exposure, leading to an increase in the IGF-IIR-G*α*q signaling pathway.

## Discussion

**Figure 8:**
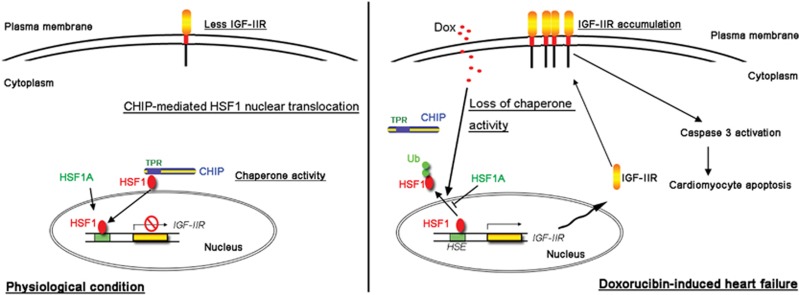
The proposed hypothesis for DOX-injured cardiomyocyte apoptosis via damage to CHIP, destabilizing HSF1 and inducing the IGF-IIR apoptosis signaling pathway. Under physiological conditions, the cytosolic CHIP is directly bound to HSF1 through its TPR domain, which stabilizes HSF1 binding to the heat shock element (HSE) on the IGF-IIR gene to inhibit its expression. Therefore, CHIP chaperones HSF1 at its TPR domain to protect cardiomyocyte apoptosis via suppressing the IGF-IIR apoptotic signaling pathway. However, DOX challenge damages the CHIP–HSF1 interaction, which in turn influences HSF1 stability and results in HSF1 degradation. Once HSF1 degrades, IGF-IIR is highly expressed and translocates into the membrane to induce caspase-3 activation, and eventually cardiomyocyte apoptosis. Moreover, supplementation with HSF1A, an HSF1 activator, alleviates DOX-induced cardiomyocyte apoptosis via suppression of the IGF-IIR apoptotic signaling pathway

In this study, we demonstrated that DOX injured cardiomyocytes via an elaborate mechanism, which increased IGF-IIR expression and resulted in cardiomyocyte apoptosis through the impairment of the ability of the CHIP chaperone to maintain HSF1 stability. CHIP stabilized HSF1 and facilitated its nuclear translocation and activity to protect cardiomyocytes. Once HSF1 instability was induced, an increase in membrane-bound IGF-IIR expression contributed to the progression of apoptosis (Figure 8). These findings provide a well-characterized mechanism for a therapeutic strategy for regulating DOX-induced heart failure through the CHIP-HSF1-IGF-IIR pathway.

In this study, we found that DOX treatment resulted in HSF1 degradation via the UPS, which upregulated IGF-IIR expression to induce cardiomyocyte apoptosis. These results are consistent with previous studies that HSF1 acts as a cardioprotective factor that mediates heat shock proteins in response to stresses in cardiomyocytes.^40, 41, 42^ However, HSF1 activation has also been reported to enhance heart failure. Vedam *et al.*^32^ indicated that DOX-induced reactive oxygen species activated HSF1 to increase its downstream Hsp25 expression, leading to heart failure. Their findings indicate that HSF1 knockout improved heart function and survival in response to DOX exposure. However, Hsp27 transgenic mice for were more resistant to DOX-induced heart failure and to ischemia/reperfusion injury *in vivo.*^43, 44^ Therefore, the role of HSF1 in cardiomyocytes is still uncertain. Our results increase the understanding of the cardioprotective role of HSF1 in cardiomyocytes.

Recent studies have shown that the CHIP can directly interact with the N-terminal domain of HSF1, facilitating HSF1 nuclear translocation.^26, 27^ These findings provide a necessary mechanism for CHIP-dependent HSF1 activation in the protection against stress-induced apoptosis. Several lines of evidence indicating that CHIP exerts its cardioprotective effect via regulating protein stability during heart failure.^23, 25^ CHIP^-/-^ mice showed increased infarct size and myocyte apoptosis after cardiac ischemia, suggesting that this chaperone function may be involved in the cardioprotective effects of CHIP.^24^ Woo *et al.*^25^ found that CHIP prevented cardiomyocyte apoptosis and cardiac dysfunction via promoting ICER destabilization as part of the IGF-I survival signaling pathway. Moreover, Naito *et al.*^23^ indicate that CHIP downregulation resulted in p53 accumulation after myocardial infarction, leading to cardiomyocyte apoptosis. It has been reported that DOX promotes the degradation of endogenous UPS substrates and activates the UPS by elevating CHIP E3 ligase activity.^37, 38^ Although Dai *et al.*^27^ indicated that CHIP had no effect on the stability and ubiquitination of HSF1, our studies show that CHIP maintained HSF1 stability via its chaperone activity. More notably, CHIP-mediated degradation activity was not involved in HSF1 instability during DOX treatment, suggesting that DOX may activate CHIP-mediated degradation activities in controlling protein quality, which led to a decrease in its chaperone activity in maintaining HSF1 stability. Supporting our findings, CHIP has been reported to stabilize protein expression either by its chaperone activity^22^ or by monoubiquitination,^39^ suggesting that CHIP prevents cardiomyocyte apoptosis via a different mechanism. Moreover, recent studies have reported that HSF1 can be ubiquitinated and degraded by the SCF complex during mitosis^45^ and the FILI-1L-hHR23A complex during heat shock.^46^ These results suggest that there are other mechanisms involved in regulating HSF1 degradation, which provide us an important subject for future investigation.

Interestingly, confocal microscopy images showed that endogenous CHIP proteins were wildly distributed in the nucleus and cytoplasm in primary NRVMs, which was inconsistent with previous findings that CHIP was primarily localized in the cytoplasm under normal conditions.^47^ In fact, CHIP partially translocates to the nucleus to maintain protein quality during cellular senescence^48^ resulting from various stresses, including oxidative stress. CHIP and HSF1 have both been reported to be associated with aging and oxidative stresses,^36, 49^ which are considered the major contributors to aging and heart failure. Therefore, our findings have significant implications for understanding heart failure and aging, and they expand our understanding of the indispensable protective role of CHIP and HSF1 in cardiomyocytes in response to stress. It is noteworthy that we found CHIP proteins to be significantly decreased in heart tissue after DOX treatment, which is inconsistent with the cell-based results. We assume that the turnover of CHIP is rapid during DOX-induced oxidative stresses, which is of interest for future investigation.

In conclusion, this study provides fundamental insights into the molecular and cellular mechanisms of DOX-induced cardiotoxicity. Our results show that the elevation of the CHIP TPR domain and HSF1 effectively reverse DOX-induced cardiomyopathy. We show that DOX increases IGF-IIR expression to trigger apoptosis via diminishing the chaperone activity of CHIP and downregulating HSF1 *in vitro* and *in vivo*. Administration of an HSF1 activator reversed DOX-induced cardiomyopathy. Taken together, our findings indicate that CHIP coordinates with HSF1 to protect cardiomyocyte against apoptosis via direct interactions that increase HSF1 stability, which provides a novel strategy for preventing the progression of heart failure.

## Materials and Methods

### Experimental animals and the administration of DOX and the small molecular drug HSF1A

All animal experiments were performed in accordance with the Guide for the Care and Use of Laboratory Animals (National Institutes of Health publication no. 85–23, revised 1996) under a protocol approved by the Animal Research Committee of China Medical University, Taichung, Taiwan. Ten-week-old Wistar Kyoto rats (WKY) were used in our experiments. The rats were housed at a constant temperature (22 °C) on a 12-h light/dark cycle with food and tap water. The animals were arranged into three groups: WKY rats (the control group), DOX rats and DOX rats treated with HSF1A (a small molecular HSF1 activator). Each group contained five animals. The DOX group was injected with DOX (5 mg/kg) for 6 consecutive weeks intraperitoneal injection to achieve a cumulative dose of 30 mg/kg, which has been well documented to achieve cardiotoxicity.^50^ The small molecular HSF1 activator HSF1A (100 mg/kg/day; Merck, Darmstadt, Germany) was injected intraperitoneally.

### Echocardiography

Echocardiographic imaging and calculations were performed according to the guidelines published by the American Society of Echocardiography using a 12 MHz linear transducer and a 5–8 MHz sector transducer (Vivid 3, General Electric Medical Systems Ultrasound, Tirat Carmel, Israel). Under ketamine (100 mg/kg, i.p.) anesthesia, measurements were made based on the M-mode and two-dimensional images obtained in the parasternal long and short axes at the level of the papillary muscles after observing at least six cardiac cycles. Interventricular septal thickness (IVS), left ventricular diameter (LVD) and left ventricular posterior wall thickness (LVPW) were measured during systole (s) and diastole (d). EF, FS and left ventricular mass and wall thickness were calculated from the M-mode images using the following formulas: (% EF =(LVDd)^3^–(LVDs)^3^/(LVDd)^3^ × 100) for the EF, (% FS=LVDd–LVDs/LVDd × 100) for the FS, left ventricular mass=1.04 × ((LVDd+LVPWd+IVSd)^3^–(LVDd)^3^) × 0.8+0.14, and relative wall thickness=2 × (LVPWd/LVDd).

### NRVM primary culture

NRVMs were prepared and cultured using a Neonatal Rat/Mouse Cardiomyocyte Isolation Kit (Cellutron Life Technology, Baltimore, MD, USA). Hearts were dissected from 1- to 3-day-old Sprague Dawley rats and transferred into a sterile beaker. Each heart was digested and stirred in the beaker at 37 °C for 12 min. The supernatant was then transferred to a new sterile tube and spun at 1200 r.p.m. for 1 min. The cell pellets were then resuspended in D3 buffer and preplated for 1 h by seeding them on an uncoated plate at 37 °C in a CO_2_ incubator to select the cardiac fibroblasts. The unattached cells were transferred to plates that had been precoated with NS medium (supplemented with 10% fetal bovine serum). After an overnight culture, the NS medium was replaced with a serum-free NW (without serum) medium. The cardiomyocyte cultures were ready for experiments 48 h after the initial plating.

### Expression plasmids and gene construction

HA-CHIP WT, HA-CHIP K30A and HA-CHIP H260Q were gifts from Dr. Lo Jeng-Fan (Robarts Research Institute and Department of Physiology and Pharmacology, University of Western Ontario, London, Ontario, Canada). Flag-HSF1 and HA-Ubiquitin were purchased from Addgene (Cambridge, MA, USA). The IGF-IIR luciferase reporter constructs were generated as previously described.^15, 16^ The pLKO.1-lentivirus constructs carrying shRNA against HSF1 and IGF-IIR, were obtained from the National RNAi Core Facility (Academia Sinica, Taipei, Taiwan).

To generate a truncated mutant of CHIP, we performed a PCR using HA-CHIP WT plasmids as the template. The primer sequences for the truncated CHIP were as follows: 5'GFP-CHIP-XhoI (nt 416–439), 5′-GTGCTCGAG**ATG**AAGGGCAAGGAGGAGAAGGAG-3' 3′GFP-CHIP-XbaI (nt 776–799), 5′-TCGAAGCTTCCGCTGCTCCTTGGCCAGGCTGTA-3′ 3′GFP-CHIP-XbaI (nt 1070–1093), 5′-TCGAAGCTTGTCTCGCTTCTTCCTCTTCTCATC-3′ 3′GFP-CHIP-XbaI (nt 1301–1324), 5′-TCGAAGCTTGTAGTCCTCCACCCAGCCATTCTC-3′ 5′GFP-CHIP-XhoI (nt 796–817), 5′-GTGCTCGAG**ATG**CGGCTGAACTTCGGGGACGAC-3′. The XhoI and XbaI sites are underlined and the initiation codon is in bold-type letters. The amplified DNA was digested with XhoI and XbaI, and the small DNA fragment was removed using the QIAquick PCR purification kit (QIAGEN, Duesseldorf, Germany). CHIP-specific inserts were subcloned into the pEGFP-N1 vector.

### Cell culture and transient transfection

H9c2 cardiomyoblast cells derived from embryonic BD1X rat heart tissue were obtained from American Type Culture Collection (ATCC, Manassas, VA, USA) and cultured in Dulbecco's modified essential medium supplemented with 10% fetal bovine serum, 2 mM glutamine, 100 U/ml penicillin, 100 mg/ml streptomycin and 1 mM pyruvate in humidified air (5% CO_2_) at 37 °C.

The cells were grown to 80% confluence on the day of transfection. The plasmids and siRNAs were transfected using the PureFection transfection reagent according to the manufacturer's instructions (System Biosciences, Palo Alto, CA, USA). All siRNAs were purchased from Sigma (St. Louis, MO, USA).

### Luciferase reporter assay

Briefly, cells were co-transfected with both luciferase IGF-IIR reporter constructs and internal control luciferase plasmids. After transfection and treatment, the cells were assayed for luciferase activity using a Dual-Glo luciferase assay system (Promega, San Luis Obispo, CA, USA). Plates were read on a Reporter Microplate Luminometer (Turner Biosystems, Sunnyvale, CA, USA). To control for potential variations in transfection or lysis efficiency, the luciferase signals were normalized to the internal control luciferase signal.

### Antibodies and reagents

The following antibodies were used in this study: anti-IGF-IIR (sc-25462, Santa Cruz Biotechnology, Dallas, TX, USA), anti-CHIP (sc-66830, Santa Cruz Biotechnology), anti-HSF1 (sc-9144, Santa Cruz Biotechnology), anti-caspase-3 (sc-7148, Santa Cruz Biotechnology), anti-p53 (sc-126, Santa Cruz Biotechnology), anti-p-p53 (Ser15, sc-101762, Santa Cruz Biotechnology), anti-HSP27 (sc-1049, Santa Cruz Biotechnology), anti-HSP70 (sc-32239, Santa Cruz Biotechnology), anti-GAPDH (sc-47724, Santa Cruz Biotechnology), anti-HA (sc-7392, Santa Cruz Biotechnology), anti-Flag (sc-807, Santa Cruz Biotechnology), anti-*β*-actin (sc-8432, Santa Cruz Biotechnology), and anti-ubiquitin (sc-8017, Santa Cruz Biotechnology); anti-Flag (#ab1162, Abcam, Cambridge, UK); and anti-PARP (#9532, Cell Signaling Technology, Danvers, MA, USA), anti-caspase-3 (#9662, Cell Signaling Technology) and anti-FLT1 (#2893, Cell Signaling Technology). All secondary antibodies (anti-rabbit, mouse and goat, HRP-conjugated antibodies) were purchased from Santa Cruz Biotechnology. All reagents were purchased from Sigma.

### Western blot analysis and immunoprecipitation

A total of 30* μ*g of total lysate or 10* μ*g of the subcellular fractions was separated using 6–12% SDS-polyacrylamide gel electrophoresis and then transferred to PVDF membranes (GE, Amersham, UK). The membranes were blocked using 5% non-fat milk and blotted with specific antibodies overnight at 4 °C. Then, the protein signals were measured using horseradish peroxidase-conjugated secondary antibodies (1:3,000 dilution, Santa Cruz Biotechnology) and Immobilon Western Chemiluminescent HRP Substrate (Millipore, Danvers, MA, USA). Densitometric analysis of the immunoblots was performed using the AlphaImager 2200 digital imaging system (Digital Imaging System, Commerce, CA, USA). The digital images were processed in Adobe Photoshop 7.0. After each analysis, each blot was stripped of antibodies using Restore Western Blot Stripping Buffer (Thermo Scientific Pierce, Rockford, IL, USA) and then incubated with the another set of antibodies. The results were analyzed and quantified using ImageJ software (NIH, Bethesda, MD, USA).

Immunoprecipitations were performed on H9c2 or NRVMs cell lysates using the PureProteome Protein G Magnetic Bead System (Millipore) according to the manufacturer's instructions.^51^ A total of 300* μ*g of cell lysate was prepared. The lysate was combined with 2* μ*g of a specific primary antibody and allowed to interact overnight while being incubated on a rotator at 4 °C. Immunoprecipitated proteins were eluted from the magnetic beads at 95 °C for 5 min and separated via SDS-PAGE. The proteins were transferred to a PVDF membrane and probed with specific antibodies.

### RNA extraction and reverse transcription-polymerase chain reaction (RT-PCR)

Total RNA was extracted using the Direct-zol RNA MiniPrep Kit (Zymo Research Corporation, Irvine, CA, USA) according to the manufacturer's instructions. Briefly, 1 *μ*g of total RNA was incubated with 0.5* μ*g of oligo dT (MD Bio., Taipei, Taiwan) at 70 °C for 15 min. Then, the RNA was mixed with buffer containing 0.25 mM dNTPs (MD Bio.), 20 U of RNasin I Plus RNase Inhibitor (Promega, San Luis Obispo, CA, USA) and 20 U of M-MLV Reverse Transcriptase (Promega) and incubated at 42 °C for 90 min to allow for cDNA synthesis. This mixture was then used for specific cDNA amplification in a GeneAmp PCR system 2400 (Perkin Elmer, Waltham, MA, USA).

### Indirect immunofluorescence and confocal microscopy

Cells were fixed with 4% paraformaldehyde for 15 min at room temperature and permeabilized with 0.1% Triton X-100 for 15 min at room temperature before staining with a specific antibody.^52^ Then, the cells were washed and stained with Alexa 546 rabbit anti-mouse IgG secondary antibodies (Invitrogen, Carlsbad, CA, USA). Images were captured using a Leica SP2 confocal spectral microscope (Leica Microsystems Inc., Buffalo Grove, IL, USA). The images were processed using Adobe Photoshop.

### Measurement of surface IGF-IIR expression

Cells were seeded into 12-well plates on the day before treatment with siRNAs or drugs. After treatment, the cells were washed with phosphate-buffered saline (PBS) and then fixed with 4% paraformaldehyde for 15 min at room temperature. The cells were then blocked with 5% goat serum and incubated with a mouse anti-IGF-IIR antibody (ab2733, Abcam) overnight at 4 °C. After staining with the primary antibody, the cells were incubated with a rabbit anti-mouse HRP-conjugated antibody for 1.5 h at room temperature. Finally, the cells were washed and incubated with the HRP substrate (1-Step Ultra TMB solution, Thermo Scientific Pierce, Rockford, IL, USA) for 30 min. The reaction was stopped using 1 M sulfuric acid. The sample was measured at 550 mm.

### Cell viability assay

Cell viability was estimated using a colorimetric assay based on the conversion of tetrazolium dye (MTT [3-(4,5-dimethylthiazol-2-yl)-2,5-diphenyltetrazolium-bromide]) into a blue formazan product. After harvesting and washing twice with PBS, the cells were cultured in phenol red-free DMEM (1 ml) with MTT (0.5 mg/ml) at 37 °C for 4 h. The cells were then incubated in isopropanol (1 ml) with shaking for 10 min, aspirated and measured spectrophotometrically at 570 nm.

### Flow cytometric analysis for caspase-3 activity

Following treatment, cells were washed with PBS, then treated with trypsin and harvested. Cells were incubated with PhiPhiLux®-G1D2 (A304R1G-5, OncoImmunin, Inc., Gaithersburg, MD, USA) at 37 °C for 30–60 min and washed by PBS before flow cytometric analysis.

### Terminal deoxynucleotidyl transferase dUTP nick end labeling (TUNEL)

After various treatments, H9c2 cells and NRVMs were fixed with a 4% paraformaldehyde solution for 30 min at room temperature. Following a rinse with PBS, the samples were incubated first with phalloidin–rhodamine for 1 h and subsequently with the TUNEL reaction mixture, containing terminal deoxynucleotidyl transferase and fluorescein isothiocyanate-dUTP (Roche Applied Science, Indianapolis, IN, USA). In heart tissues, 3-μm-thick paraffin sections were deparaffinized by immersion in xylene, then rehydrated, and incubated in PBS with 2% H_2_O_2_ to inactivate endogenous peroxidases. Next, the sections were incubated with proteinase K (20* μ*g/ml), washed in PBS, and incubated with terminal deoxynucleotidyl transferase for 90 min and with fluorescein isothiocyanate-dUTP for 30 min at 37 °C using an apoptosis detection kit (Roche Applied Science). Then, the sections were stained with 4, 6-diamidino-2-phenylindole to detect cell nuclei via UV light microscopic observations (blue). The samples were analyzed in a drop of PBS under a fluorescent and UV light microscope in this state using an excitation wavelength in the range of 450–500 nm, with detection in the range of 515–565 nm (green). The number of TUNEL-positive cardiac myocytes was determined by counting 3 × 10^5^ cardiac myocytes. All morphometric measurements were performed by at least two individuals independently in a blinded manner.

### Statistical analysis

All experiments were performed at least three times. Statistical analyses were performed using the GraphPad Prism5 statistical software (San Diego, CA, USA). Statistical significance was set at *P*<0.05. Multiple comparisons of the data were analyzed via ANOVA. All results were quantified by ImageJ software (NIH, Bethesda, MD, USA) and processed using Adobe Photoshop.

## Figures and Tables

**Table 1:** Echocardiographic assessments of cardiovascular function

	**Control group (*n*=5)**	**Dox group (*n*=5)**	**Dox/HSF1A group (*n*=5)**
IVSd (mm)	1.19±0.08	1.01±0.02*	1.22±0.12^##^
LVIDd (mm)	8.27±0.72	7.63±0.56**	7.63±0.24
LVPWd (mm)	1.22±0.17	0.85±0.06**	1.16±0.15^#^
IVSs (mm)	2.33±0.06	1.99±0.31	2.43±0.28^#^
LVIDs (mm)	5.62±0.30	4.26±0.37**	4.36±0.26
LVPWs (mm)	2.21±0.08	1.62±0.42*	2.04±0.18
EDV (Teich)	1.33±0.18	0.91±0.13*	0.95±0.21
ESV (Teich)	0.44±0.16	0.19±0.05**	0.24±0.03
EF (Teich)	76.85±2.36	64.75±7.10*	76.36±2.29^#^
%FS	40.70±2.07	29.38±2.66***	40.15±2.04^###^
LVd mass (ASE)	1.15±0.06	0.96±0.02**	1.01±0.09
LVs mass (ASE)	1.23±0.1	1.04±0.02	1.14±0.03

Abbreviations: EF, ejection fraction; EDV, end-diastolic volume; ESV, end-systolic volume; FS, fractional shortening; IVSd, interventricular septum at diastole; IVSs, interventricular septal thickness in systole; LVIDd, left ventricular diameter in diastole; LVIDs, left ventricular diameter in systole; LVPWd, left ventricular posterior wall thickness at diastole; LVPW, left ventricular posterior wall thickness; LVd and LVs mass, left ventricular mass during diastole and systole, respectively

Values are mean±S.D. *P<0.05, **P<0.01 and ***P<0.001 are compared with control group; ^#^*P*<0.05, ^##^*P*<0.01 and ^###^*P*<0.001 are compared with Dox group. Values are the means±S.D.

**Table 2:** Cardiac characteristics of the control, DOX and DOX/HSF1A groups

	**Control group (*n*=5)**	**Dox group (*n*=5)**	**Dox/HSF1A group (*n*=5)**
Whole heart weight (WHW), g	1.10±0.07	1.11±0.01	1.08±0.11
Left heart weight (LHW), g	0.79±0.01	0.69±0.01	0.81±0.10^#^
Tibia (mm)	41.3±0.28	40.91±0.13	41.13±0.30
LVW/WHW	0.72±0.03	0.63±0.01*	0.75±0.02^##^
WHW/tibia, g/mm	0.0265±0.00174	0.02709±0.00039	0.02631±0.00262
LVW/tibia, g/mm	0.01934±0.00011	0.01688±0.00024*	0.01972±0.00238^#^

Abbreviations: LVW, left ventricular weight; LVW/tibia, left ventricular weight normalized by tibial length; LVW/WHW, left ventricular weight normalized by whole heart weight; WHW, whole heart weight; WHW/tibia, whole heart weight normalized by tibial length

Values are mean±S.D. *P<0.05, compared with control group; ^#^*P*<0.05 and ^##^*P*<0.01, compared with Dox group. *indicates a significant difference from the control group at *P*<0.05. ^#^indicates a significant difference from the DOX group at *P*<0.05. ^##^indicates a significant difference from the DOX group at *P*<0.01

## Abbreviations

CHIP: carboxyl-terminus of *Hsp70* interacting protein
IGF-IIR: insulin-like growth factor receptor II
HSF1: heat shock transcription factor 1
Dox: doxorubicin
UPS: ubiquitin-proteasome system
FS: fractional shortening
EF: ejection fraction
