# Supplementary material for: Doxorubicin attenuates CHIP-guarded HSF1 nuclear translocation and protein stability to trigger IGF-IIR-dependent cardiomyocyte death
Source: Cell Death Dis. 2016 Nov 3;7(11):e2455–. doi: 10.1038/cddis.2016.356 (PMC5260882; doi:10.1038/cddis.2016.356)
Supplement: Supplementary Information [file cddis2016356x1.docx]

**Supplementary information**

**Doxorubicin attenuates CHIP-guarded HSF1 nuclear translocation and protein stability to trigger IGF-IIR-dependent cardiomyocyte death**

Chih-Yang Huang^1^,Wei-Wen Kuo^2^, Jeng-Fan Lo^3^,Tsung-Jung Ho^4,5^, Pei-ying Pai^6^, Shu-Fen Chiang^7^, Pei-Yu Chen ^8^, Fu-Jen Tsai^4^, Chang-Hai Tsai^9^,Chih-Yang Huang^4,10,11^

^1^Translation Research Core, China Medical University Hospital, China Medical University, Taichung

^2^Department of Biological Science and Technology, China Medical University, Taichung, Taiwan

^3^Institute of Oral Biology, National Yang-Ming University, Taipei, Taiwan

^4^Chinese Medicine Department, China Medical University Beigang Hospital, Taiwan

^5^School of Chinese Medicine, China Medical University, Taichung, Taiwan

^6^Division of Cardiology, China Medical University Hospital, Taichung, Taiwan

^7^Cancer Center, China Medical University Hospital, Taichung, Taiwan.

^8^Department of pathology, China Medical University Hospital, Taichung, Taiwan

^9^Department of Healthcare Administration, Asia University, Taichung, Taiwan

^10^Graduate Institute of Basic Medical Science, China Medical University, Taichung

^11^Department of Health and Nutrition Biotechnology, Asia University, Taichung


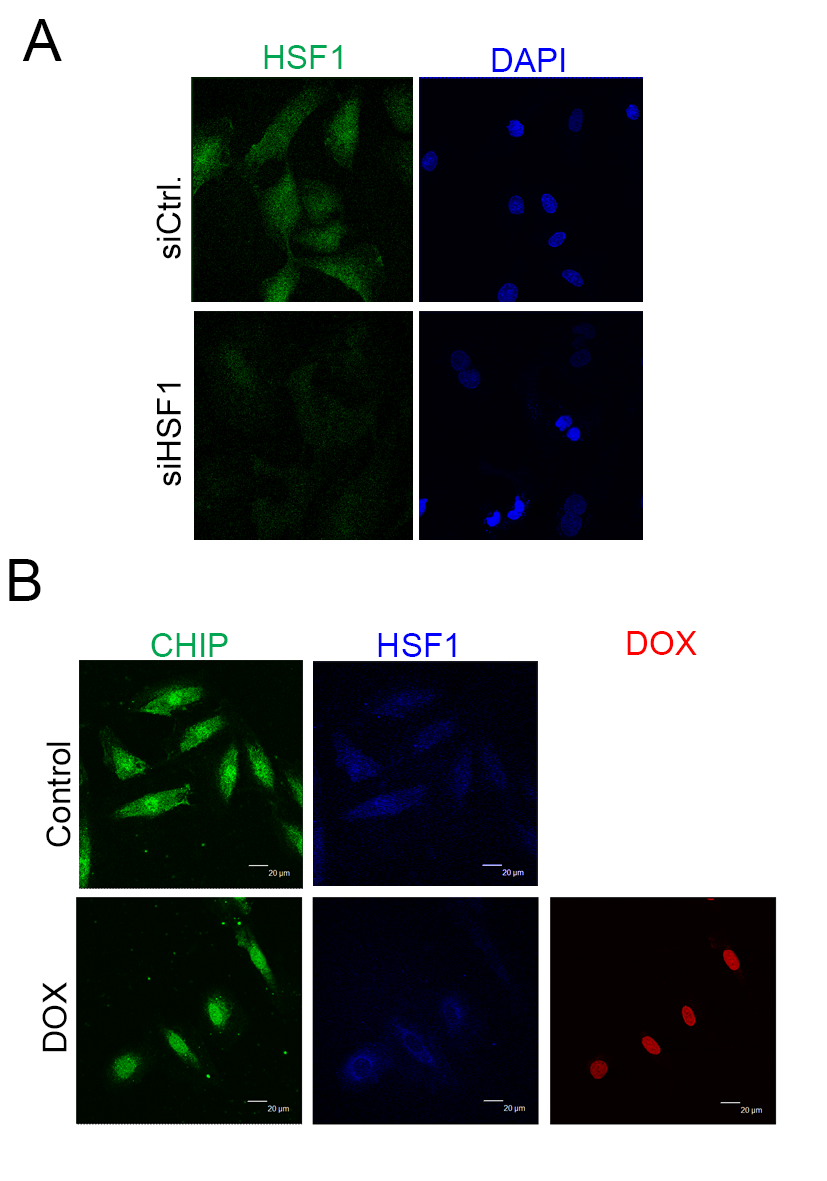


**Fig. S1. DOX treatment blocked nuclear translocation of HSF1.**

(A) NRVMs were transfected with siRNA for 48 hrs. Cells were fixed and stained with antibodies against HSF1 by immunofluorescence. These results confirmed the specificity of HSF1 antibodies. (B) NRVMs were treated with 1μM doxorubicin for 24 hrs. Cells were fixed and stained with antibodies against HSF1 and CHIP by immunofluorescence.


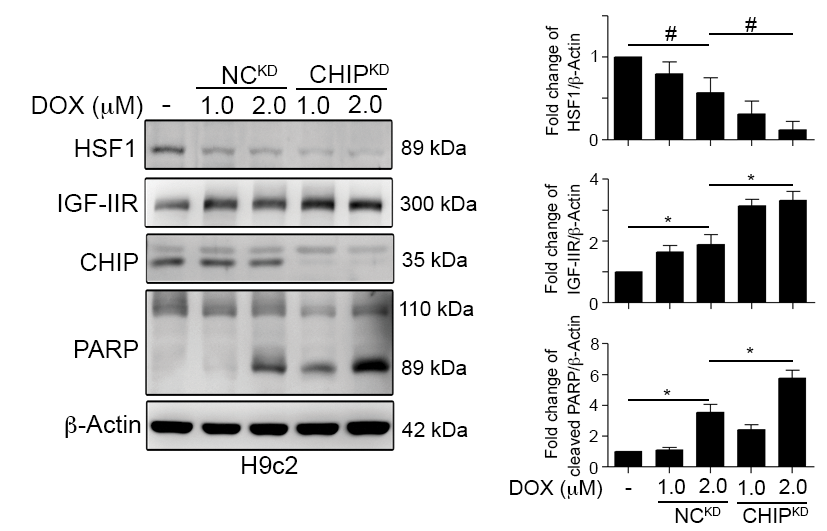


**Fig. S2. Deficiency of CHIP promoted HSF1 instability and cardiomyocyte apoptosis.**

H9c2 cells were infected with lentiviral plasmids carrying scrambled or CHIP shRNA. After H9c2-scramble^KD^ and H9c2-CHIP^KD^ cell lines were established, the cell lines were treated with 1 or 2 μM DOX for 24 hrs. HSF1, IGF-IIR and PARP were estimated via immunoblotting. Quantification of these results is shown right (n=3). *P<0.05 and #P<0.05.


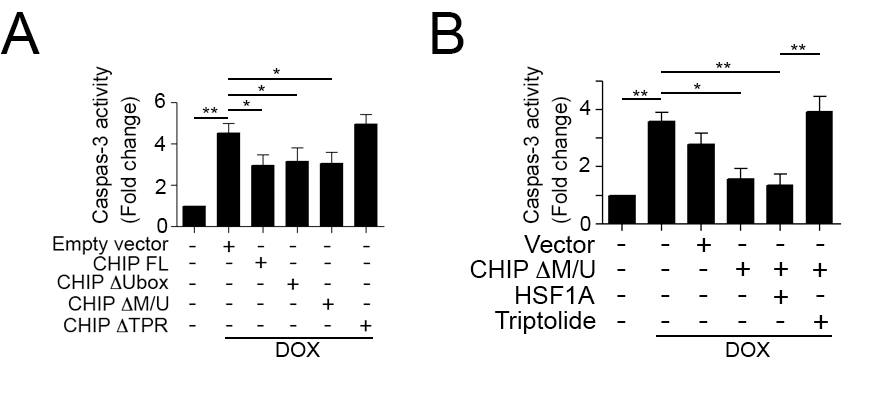


**Fig. S3. CHIP-ΔM/U clearly alleviated DOX-induced caspase-3 activity.**

(A) Primary NRVMs were transfected with different CHIP deletion mutant constructs for 24 hrs and then treated with 1 μM DOX for 24 hrs. The caspase-3 activities were measured by flow cytometry. Quantification of caspase-3 activity is shown below (n=3). *P<0.05 and **P<0.01.

(B) Primary NRVMs were transfected with CHIP-ΔM/U-GFP for 24 hrs. Cells were then treated 10 μM HSF1A or 1 μM triptolide with 1 μM DOX for 24 hrs. Quantification of caspase-3 activity is shown below (n=3). *P<0.05 and **P<0.01.
